# Omicron spike protein: a clue for viral entry and immune evasion

**DOI:** 10.1038/s41392-022-01193-7

**Published:** 2022-09-28

**Authors:** G. M. N. Behrens, A. Cossmann, M. Hoffmann

**Affiliations:** 1 Department for Rheumatology and Immunology, Hannover Medical School, Hannover, Germany; 2 German Centre for Infection Research, partner site Hannover-Braunschweig, Hannover, Germany; 3 CiiM, Centre for Individualized Infection Medicine, Hannover, Germany; 4 Infection Biology Unit, German Primate Center, Göttingen, 37077 Germany; 5 Faculty of Biology and Psychology, Georg-August-University Göttingen, Göttingen, Germany

**Keywords:** Vaccines, Infectious diseases

In a recent study in *Science*, Bowen et al.^1^ report that mutations in Omicron sublineage’s spike protein enhance ACE2 binding, impair fusogenicity, and dampen the neutralizing activity of antibodies after vaccination or infection. The data describe how Omicron and especially the BA.5 variant evade neutralizing antibody responses and argue for using mRNA booster vaccination to increase immunity.

The SARS-CoV-2 Omicron variant (Pango lineage B.1.1.529) is genetically very different from the ancestral virus (Wuhan-Hu-1, Pango lineage B). It accounts for almost all infections during the first six months of 2022. Omicron comprises several sublineages and BA.5 now replaced the previously dominant sublineages BA.1, BA.2, and BA.2.12.1.

The SARS-CoV-2 spike (S) protein is crucial for cell entry, pathogenicity, and is the key target of the adaptive immune response. For SARS-CoV-2 attachment, the S protein binds to the cellular protein angiotensin-converting enzyme 2 (ACE2).^2^ Subsequently, the S protein is primed by host cell proteases and drives the fusion of the viral and cellular membranes, which can take place at the plasma membrane or within endo-/lysosomes. Next, the viral genome is released into the cytoplasm, and translation of viral proteins and genome replication start. The SARS-CoV-2 S protein also is a pathogenicity factor. S protein expression on the cell surface leads to fusion with neighboring cells and the formation of giant cells with multiple nuclei, so-called syncytia, which were observed in postmortem biopsies from patients that died from COVID-19. Finally, the SARS-CoV-2 S protein is the main target of neutralizing antibodies, which are correlated for protection against SARS-CoV-2 infection and severe COVID-19.

**Fig. 1:**
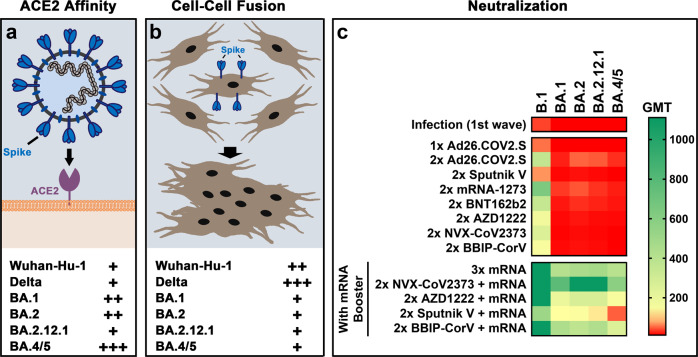
Higher binding affinity, lower syncytium formation, and more immune escape by Omicron. **a** The RBDs of BA.1, BA.2 and BA.4/5 have an about 2–6-fold higher affinity for ACE2 than the RBDs of the SARS-CoV-2 isolate Wuhan-Hu-1 and the delta variant. **b** The S proteins of all Omicron sublineages are less capable to fuse cells compared to the S proteins of Wuhan-Hu-1 or Delta, the latter of which is known to possess high cell-cell fusion capacity. **c** The polyclonal plasma neutralizing antibody responses (geometric mean titers, GMT as described in Bowen et al.^1^) for Omicron sublineages after homologous prime/boost vaccination showed low titers and thus significant immune escape when compared to neutralization against ancestral virus Wuhan-Hu-1 (B.1). The vaccines studied were mRNA-based (Moderna mRNA-1273, Pfizer BNT162b2), vector-based (Janssen Ad26.COV2.S, AstraZeneca AZD1222, Gamaleya National Center of Epidemiology and Microbiology Sputnik V), protein-based (Novavax NVX-CoV2373), and inactivated virions (Sinopharm BBIP-CorV). Triple mRNA-based vaccination resulted in robust neutralization titers comparable to heterologous mRNA booster, which also led to appreciable neutralization titers. Plasma antibodies from individuals infected during the first wave showed little neutralization against Omicron

The S proteins of the Omicron sublineages harbor multiple mutations, many of them reside in the RBD and represent escape mutations that reduce the ability of antibody binding. While some RBD mutations are known to augment ACE2 binding, most of them weaken spike-ACE2 interaction. Thus, one would assume that Omicron sublineages would have reduced ACE2 affinity. However, the opposite is true. Using biolayer interferometry and surface plasmon resonance analyses, Bowen *et al*. show that the RBDs of BA.1, BA.2 and BA.4/5 (identical on protein sequence) have a ~2–6-fold higher affinity for ACE2 than the RBDs of the SARS-CoV-2 isolate Wuhan-Hu-1 and the delta variant (Fig. 1a). Interestingly, the RBD of BA.2.12.1, which differs from the BA.2 RBD by only one mutation, has a much lower ACE2 affinity than BA.2 RBD, indicating that this difference is due to mutation L452Q. But why do the RBDs of Omicron sublineages have high affinities for ACE2 despite harboring several mutations that weaken ACE2 interaction? The answer is provided by another study, which revealed that some Omicron-specific RBD mutations establish new ACE2 contacts and thus compensate for RBD mutations that weaken ACE2 interaction.^3^

The SARS-CoV-2 S protein also induces cell-cell fusion when expressed on the cell surface. Thus, differences in the capability of SARS-CoV-2 variants to cause syncytium formation may in part reflect their pathogenic potential. Using a cell-cell fusion assay, Bowen et al. demonstrate that the S proteins of all Omicron sublineages tested are less capable to fuse cells compared to the S proteins of Wuhan-Hu-1 or Delta, the latter of which is known to possess high cell-cell fusion capacity (Fig. 1b). Thus, with respect to causing tissue damage by syncytium formation, Omicron sublineages appear to have lower pathogenic potential than previous SARS-CoV-2 variants.

Previous work about triple vaccination revealed expansion of pre-existing and *de novo* induction of memory B cells specific for the SARS-CoV-2 S protein, leading to antibodies with enhanced neutralization potency and breadth against variants. In addition, most monoclonal antibodies showed a substantial drop or even a complete loss of activity against Omicron sublineages. The work by Bowen and coworkers adds to this by investigating the plasma neutralizing activity elicited by different vaccine platforms or by vaccinating inactivated virions. The researchers used pseudotyped vesicular stomatitis virus (VSV) with S proteins harboring the Omicron mutations to determine immune evasion. They compared the homologous prime/boost schemes to triple mRNA vaccination or heterologous booster, when after vector-, protein- or inactivated virion-based homologous vaccinations mRNA vaccines were used for the third shot. The overall results show a significant evasion of plasma neutralizing antibody responses against Omicron sublineages when compared to neutralization against ancestral virus (Fig. 1c). However, triple mRNA-based vaccination or heterologous mRNA booster resulted in robust neutralization titers and little immune evasion expressed as fold-change neutralization reduction. The immune escape especially by BA.4/5 was most prominent after the heterologous booster compared to triple mRNA vaccination, which may be mechanistically interesting but less relevant for vaccine efficacy. One should also be cautious when comparing the individual vaccine regimens, since the cohorts are limited in size, some varying for time intervals between vaccination and blood withdrawal, and others slightly heterogeneous for vaccine regimens.

There is currently great interest in elucidating differences in the strength and breadth of the neutralizing antibody response elicited by only infection compared to responses induced by “hybrid immunity”, namely infection and vaccination. Post-vaccination Omicron BA.1 breakthrough infection leads to strong neutralizing activity against Omicron BA.1, BA.2, and earlier SARS-CoV-2 variants of concern, but not against Omicron sublineages BA.4 and BA.5. It seems that BA.1 breakthrough infections prompted a vigorous recall response, mostly expanding memory B cells against epitopes shared broadly amongst variants.^4^ Preliminary data indicate, however, that a third vaccination six months after the initial shots did not improve neutralization potency or breadth among convalescent individuals after COVID-19.^5^

Thus, the increasing number of vaccinations, vaccine breakthrough or reinfections will add more complexity to COVID-19 research and for providing advice how to place adapted mRNA vaccines, which incorporate an Omicron variant strain. For now, the study by Bowen et al. suggests that despite the fact that Omicron sublineages escape polyclonal neutralizing antibody responses after a primary vaccine series, vaccine boosters most likely offer sufficient protection against Omicron-associated severe illness.
